# Electrochemistry and Spectroscopy-Based Biosensors

**DOI:** 10.3390/bios13010009

**Published:** 2022-12-22

**Authors:** Gintautas Bagdžiūnas

**Affiliations:** 1 Group of Supramolecular Analysis, Institute of Biochemistry, Life Sciences Centre, Vilnius University, Saulėtekio Av. 7, LT-10257 Vilnius, Lithuania; gintautas.bagdziunas@gmc.vu.lt; 2 Department of Functional Materials and Electronics, Center for Physical Sciences and Technology, Saulėtekio Av. 3, LT-10257 Vilnius, Lithuania

During and after the COVID-19 pandemic, the development of low-cost detection and analysis methods of bioanalytes as well as infection biomarkers became an increasingly important challenge in order to improve public and personal health. This challenge is implied in the elaboration of biomaterials and instruments to thrive in achieving biosensing objectives. In turn, to achieve and mimic the high selectivity found in nature, functional biomolecules such as enzymes, antibodies, and deoxyribo- (DNA) as well as ribonucleic acid (RNA) as sensitive biological recognition elements have successfully been integrated into biosensors. First of all, biological elements are responsible for the detection of analytes by changing the optical or electrochemical properties of the recognition element. Secondly, to read and transform one signal into another one, the integration of a detector element is needed in the devices. Therefore, optical, electrochemical, and spectrofluorometric detector elements have often been applied to engineer biosensors. This Special Issue in *Biosensors*, titled “Electrochemistry and Spectroscopy-Based Biosensors”, compiles the recent research in the field of electrochemical and optical biosensors. This Special Issue consists of nine papers and one review that cover fundamental and practical studies on biosensors.

This themed issue highlights some important trends in vibrational-spectroscopy-based biosensors. Mousavi, with colleagues, summarized the recent advances in the diagnosis of inflammation using graphene-quantum-dot-based surface-enhanced Raman spectroscopy (SERS) [1]. In this review, the method of graphene quantum dot (GQD) synthesis and the detection mechanisms of inflammation by SERS have been described by the authors. Another detection phenomenon widely used by the authors of this Special Issue was fluorescence. It is well-known that fluorescent biosensors have become a research hotspot due to their simple operation, low cost, short time, high sensitivity, and high specificity. Sun et al. fabricated a ratiometric fluorescent biosensor based on multi-walled carbon nanotubes@gold nanoclusters (MWCNTs@Au NCs) and duplex-specific nuclease-assisted signal amplification applied for the detection of colorectal-cancer-associated microRNA (miR-92a-3p) extracted from exosomes [2]. Furthermore, an innovative whispering gallery mode microcavity immunosensor based on a prefab hollow glass microsphere with liquid crystal sensitization has successfully been demonstrated by Niu et al. for the detection of label-free cardiac troponin complexes [3]. Talaat and co-authors employed selective spectrofluorometric sensing of darolutamide and thalidomide as non-steroidal antiandrogens in pharmaceutical preparations and biofluids [4].

Another part of this themed Special Issue is based on electrochemical biosensors, which can be used to analyze analytes through the direct conversion of biochemical reactions into electronic signals. It is worth mentioning that third-generation electrochemical biosensors based on the direct electron (DET) or hole-transfer-type enzymes are a promising path towards practical biosensors. However, such enzymes are very rare or unstable on electrodes. Therefore, in our group an innovative enzyme functionalization strategy in organic solvents was employed to develop biosensing monolayer-based platforms, for the detection of glucose, utilizing the quasi-DET mechanism [5]. The colleagues from our department have demonstrated the bioelectrocatalytic oxidation of D-tagatose based on the DET using immobilized and variously oriented fructose dehydrogenase on three different bioelectrode surfaces [6]. Thompson and Lai successfully designed a uranyl ion (UO2+)-specific peptide with a 12-amino acid sequence and used it in the fabrication of an electrochemical sensor [7]. Alacid et al. developed an easy-to-use portable electrochemical biosensor to measure the activity in situ and in real-time of the alkaline phosphatase enzyme to identify the presence of its inhibitors [8]. Moreover, a sensor for the determination of Fe^2+^ and/or Fe^3+^ ions, as important ions in biochemistry and medicine, using a polyaniline layer as an ion-to-electron transducer, as well as chelating molecules has been introduced by Ismail et al. [9]. Pham, with colleagues, reported a systematic investigation to identify the causes of the signal instability of the chemical vapor deposition of a MoS2-based field-effect transistor (FET) as an electrochemical detecting element of their biosensors, in addition to developing a strategy to mitigate the effects of the physical degradation of the device and enhance the overall device stability [10]. Taken together, all of these different biosensors can be applied in practice for the detection of bioanalytes as disease biomarkers.

The Guest Editor would like to thank all of the authors for their submissions to this Special Issue, as well as all of the reviewers for helpful discussions and helping to ensure the quality of the submitted papers. Additionally, I thank the staff at the Editorial Office of *Biosensors* for their efficient assistance.
